# Ghrelin Through GHSR1a and OX1R Heterodimers Reveals a Gαs–cAMP-cAMP Response Element Binding Protein Signaling Pathway *in Vitro*

**DOI:** 10.3389/fnmol.2018.00245

**Published:** 2018-07-17

**Authors:** Qingjie Xue, Bo Bai, Bingyuan Ji, Xiaoyu Chen, Chunmei Wang, Peixiang Wang, Chunqing Yang, Rumin Zhang, Yunlu Jiang, Yanyou Pan, Baohua Cheng, Jing Chen

**Affiliations:** ^1^Neurobiology Institute, Jining Medical University, Jining, China; ^2^Department of Pathogenic Biology, Jining Medical University, Jining, China; ^3^Department of Physiology, Taishan Medical University, Taian, China; ^4^Division of Biomedical Sciences, Warwick Medical School, University of Warwick, Coventry, United Kingdom

**Keywords:** growth hormone secretagogue receptor 1α (GHSR1a), orexin type 1 receptor (OX1R), heterodimerization, allosteric signaling, neuroblastoma cell proliferation

## Abstract

Growth hormone secretagogue receptor 1α (GHSR1a) and Orexin 1 receptor (OX1R) are involved in various important physiological processes, and have many similar characteristics in function and distribution in peripheral tissues and the central nervous system. We explored the possibility of heterodimerization between GHSR1a and OX1R and revealed a signal transduction pathway mechanism. In this study, bioluminescence and fluorescence resonance energy transfer and co-immunoprecipitation (Co-IP) analyses were performed to demonstrate the formation of functional GHSR1a/OX1R heterodimers. This showed that a peptide corresponding to the 5-transmembrane domain of OX1R impaired heterodimer construction. We found that ghrelin stimulated GHSR1a/OX1R heterodimer cells to increase the activation of Gαs protein, compared to the cells that express GHSR1a. Stimulation of GHSR1a/OX1R heterodimers with orexin-A did not alter GPCR interactions with Gα protein subunits. GHSR1a/OX1R heterodimers induced Gαs and downstream signaling pathway activity, including increase of cAMP-response element luciferase reporter activity and cAMP levels. In addition, ghrelin induced a higher proliferation rate in SH-SY5Y cells than in controls. This suggests that ghrelin GHSR1a/OX1R heterodimers promotes an upregulation of a Gαs-cAMP-cAMP-responsive element signaling pathway *in vitro* and an increase in neuroblastoma cell proliferation.

## Introduction

Ghrelin, a 28-amino acid peptide, is the first endogenous ligand for GHSR1a. GHSR1a couples to Gαq resulting in activation of phospholipase C (PLC), inositol trisphosphate (IP3), and mobilization of Ca^2+^ (Holst et al., 2004). Apart from the pituitary gland, GHSR1a is distributed in the central nervous system (CNS) widely, especially in hypothalamic regions (Howick et al., 2017; Medrano et al., 2017). GHSR1a is also expressed in the heart, pancreas, intestine, kidney, adipose tissue, and the male and female reproductive systems. GHSR1a is important in many processes, including stress, reward and feeding behavior. Recent data have revealed the dimerization of various receptors, indicating their ability to interact with GHSR1a in signal regulation, changes in the signal cascade, and changes in the trafficking and internalization of both protomers of the dimer complex (Howick et al., 2017). Furthermore, the data show that GHSR1a and other receptors, including dopamine 1 receptor (D1R), dopamine 2 receptor (D2R) (Kern et al., 2012), melanocortin-3 receptor (MC3R) (Rediger et al., 2011), serotonin 2C receptor (5-HT2C) (Schellekens et al., 2015), and cannabinoid type 1 receptor (CB1) (Kola et al., 2005), can dimerize with GHSR1a. After dimerization, these receptor dimers modify, assemble, and block existing signaling pathways, leading to different physiological functions; thus, the dimerized growth hormone secretagogue system appears to play an important and complex role (Wellman and Abizaid, 2015).

Orexin-A and Orexin-B are neuropeptides secreted by neurons (Sakurai et al., 1998). They bind to orexin receptor 1 (OX1R) and 2 (OX2R). Orexin-A binds OX1R preferentially. It can also bind to OX2R. However, orexin B binds OX2R only. The OX1R is a Gαq protein-coupled receptor that classically induces PLC activation and subsequent cellular calcium transients. OX1R also stimulates cAMP synthesis in primary rat astrocyte culture (Woldan-Tambor et al., 2011). OX2R is primarily associated with the Gαq, Gαi and Gαs subtypes (Tang et al., 2008; Chen et al., 2015; Bai et al., 2017). Orexin receptors are widely expressed in the CNS where orexin neuron terminals are located. Recently, orexin neurons were found to play an essential part in regulating various behavioral and physiological responses mediated by the hypothalamus, such as in circadian rhythms (Gooley et al., 2006; Chen et al., 2015), anxiety, cardiorespiratory function (Mcdowall et al., 2006), feeding (Inutsuka et al., 2014), and reward (Gutierrez et al., 2011). GPCRs form heterodimers as well as homodimers. Human OX1R has been found to homodimerize and interact with other receptors functionally. For example, GPR103 and OX1R heterodimerize, as do OX1R and kappa opioid receptors (κOR) (Chen et al., 2015; Davies et al., 2015; Bai et al., 2017). GPCR heterodimers can exhibit novel function or elevate its own activity. Heterodimerization is important for their response and trafficking, and many important physiological processes take place through the interactions of these receptors.

GPCRs play an essential role in cell communication. Although they form functional monomers, there is growing evidence that GPCR dimerization plays a key role in the cooperation of cell signal integration (Chen et al., 2015). GHSR1a and OX1R are involved in stress, reward and feeding behavior. This supports the scientific hypothesis that dimerization of GHSR1a/OX1R may change their original functions, which readjust their reaction to ghrelin and play an important role in the ghrelin system and hypothalamus. Our study aimed to detect this possibility and survey a heterodimerization-mediated signal transduction mechanism. We also explored whether this type of signal transduction is involved in the proliferation of neuroblastoma cells.

## Experimental Procedures

### Plasmids, Reagents, Antibodies

The mammalian expression vector pcDNA3.1(+) was purchased from Invitrogen Life Technologies (Paisley, United Kingdom). The plasmids pcDNA3.1-OX1R, which contain human OX1R cDNA, and pcDNA3.1-GHSR1a, which contains GHSR1a, were obtained from the cDNA Resource Centre (Bloomsburg University, Bloomsburg, PA, United States). pEYFP-N1, containing EYFP, and pRluc-N1, containing the Rluc plasmid, were provided by Clontech and PerkinElmer, Inc. SRE-luc, CRE-luc and NFAT-RE-luc were obtained from Promega (Madison, WI, United States).

Human ghrelin and orexin-A were purchased from Phoenix Pharmaceuticals (Belmont, CA, United States). HEPES-buffered phenol red-free medium, DMEM (Dulbecco’s Modified Eagle Medium) and RPMI-1640 culture medium were purchased from Gibco, (Gibco, Invitrogen, Paisley, United Kingdom). Forskolin (FSK) was purchased from Sigma-Aldrich Shanghai Trading Co. Ltd. (Shanghai, China). Anti-Myc, Anti-HA, Anti-GHSR1a and anti-OX1R antibodies were obtained from Novus Biologicals (Abingdon, United Kingdom). Anti-HA-agarose was purchased from Pierce Chemical Co.

### Construction of Expression Vectors

GHSR1a-EYFP, CRH1R-ECFP and OX1R-ECFP, which respectively encode an ECFP-tag and EYFP-tag were constructed. Enhanced cyan fluorescent protein (ECFP) and EYFP were attached to the C-termini of OX1R and GHSR1a by inserting the ORFs of OX1R, CRH1R and GHSR1a into the ECFP-N1 and EYFP-N1 vectors, respectively, resulting in OX1R-ECFP, CRH1R-ECFP and GHSR1a-EYFP. Insertion was confirmed by enzyme digestion and sequencing. Gαi2-Rluc8, Gαq-Rluc8, Gαs-Rluc8, Myc-GHSR1a and HA-OX1R were constructed as described previously (Chen et al., 2015), to generate Myc-tagged GHSR1a or HA-OX1R, sequences encoding the Myc epitope tag (EQKLISEEDL). The HA epitope tags (YPYDVPDYA) were PCR-inserted at the N-terminus of GHSR1a or OX1R. Fusing either the N-terminal fragment of Venus (Venus N: amino acids 1–172) or the C-terminal fragment of Venus (Venus C: amino acids 156–239) was used to produce GHSR1a-VN173 and OX1R-VC155. OX1R-Rluc and CRH1R-Rluc were constructed as described; all of these constructs encode a C-terminal Rluc tag. All reconstructed plasmids were verified by commercial DNA sequencing.

### Cell Culture and Transfection

The HEK293 and SH-SY5Y cell lines were cultured in DMEM culture medium at 37°C in 5% CO_2_. When the cell fusion density was approximately 80% (cell fusion during transfection varied during different experiments), cells were transfected using Lipofectamine 2000. After transfection for 6 h, the serum-containing medium was replaced, and cells were cultured for 24 h. Plasmid expression was observed via fluorescence microscopy. Empty vectors were added to normalize the final plasmid amount. HEK293 cells were transfected with pcDNA3.1-OX1R, pcDNA3.1-GHSR1a and pcDNA3.1-OX1R/pcDNA3.1-GHSR1a to obtain the next generation of cells stably expressing GHSR1a, OX1R and GHSR1a/OX1R. Screening was performed in 24-well plates with G418 (0.5 mg/mL) (Gibco, Invitrogen, United Kingdom) for 10 weeks. Receptor expression was assessed by performing Western blotting.

### Confocal Microscopy

HEK293 cells were transfected with GHSR1a-EYFP and OX1R-ECFP plasmids. The cells were transferred to glass cover slips in 6-well plates after 24 h. The glass cover slips previously underwent high-pressure sterilization and were treated with Poly-D-lysine at 0.05 mg/mL for 3 h, followed by washing 3 times with PBS. After culture for 24 h at 37°C, the cells were washed with PBS for 3 times and were then fixed for 5 min. The cells were washed with PBS 3 times (5 min each). A Leica DMRE laser scanning confocal microscope (Leica, Milton Keynes, United Kingdom) was used to detect fluorescence as previously described (Chen et al., 2015; Bai et al., 2017).

### BRET Assay

To monitor constitutive GHSR1a and OX1R interactions, OX1R-Rluc and GHSR1a-EYFP plasmids were transfected into HEK293 cells at ratios of 1:1, 1:2, 1:3, 1:4, 1:5, 1:6, and 1:8 to express donor protein with increasing amounts of acceptor protein. At 24 h post-transfection, cells were trypsinized and cultured on microplate for another 24 h. Coelenterazine h (5 μM; Promega) was added for BRET measurements with an Rluc filter (480 nm) and an EYFP filter (550 nm). To monitor the induced interactions between GHSR1a and OX1R, BRET signals were measured, with slight modifications. Lastly, cells were transfected with OX1R-Rluc and/or GHSR1a-EYFP and stimulated with an agonist (ghrelin or orexin-A). The experimental assay method was carried out as previously described (Chen et al., 2015) using the BRET saturation assay. HEK293 cells were co-transfected with a constant amount of the OX1R-Rluc construct, each at 0.15 μg/well, and increasing amounts of the EYFP construct (0.15–1.2 μg/well). Calculated BRET ratios were plotted relative to total fluorescence/luminescence ratios, and the data were analyzed by non-linear regression curve fitting (one site–specific binding) using GraphPad Prism software.

### FRET Assay

Fluorescence resonance energy transfer (FRET) assays were performed as described previously (Ma et al., 2007; Cai et al., 2017). The EYFP-GHSR1a and ECFP-OX1R plasmids were co-transfected into HEK293 cells as the FRET system. In addition, donor ECFP-OX1R and acceptor EYFP, ECFP and EYFP-GHSR1a, or ECFP-CRH1R and EYFP-GHSR1a were co-transfected as negative controls. FRET signals were detected using a FLUOstar OPTIMA microplate reader (BMG Labtech, Germany) after 20 h. The calculation method and equation used are described previously (Chen et al., 2015; Bai et al., 2017).

Calculation of FRET efficiency: After 12–24 h, FRET signals were detected using the FRET Kit for the Leica AM TIRF MC system (Leica Microsystems). For each experiment, at least 10 live cells were analyzed for each condition (donor ECFP-OX1R or acceptor EYFP-GHSR1aor ECFP-OX1R and EYFP-GHSR1a). The efficiency of FRET (EA (i)) was calculated using the following equation:

$$\text{EA}(\text{i})=\frac{\text{B-A}\times \beta -\text{C}\times (\gamma -\alpha \times \beta )}{\text{C}\times (1-\beta \times \delta )}$$

Where A, B, and C correspond to the intensities of the three signals (donor, FRET, and acceptor, respectively), and α, β, γ, and δ are the calibration factors generated by the acceptor- and donor-only references.

### Co-immunoprecipitation (Co-IP) and Immunoblotting

Co-immunoprecipitation was performed as previously described (Wang et al., 2014). The HA-OX1R and Myc-GHSR1a plasmids were co-transfected into HEK293. After 36 h, the medium was discarded, and the HEK293 cells were washed with 1 mL of precooled PBS for twice and centrifuged at 3000 r/min for 3 min. The cells were collected and 200 μL of weak lysate buffer was added (RIPA including PMSF) for cracking at 4°C. The supernatant was removed after centrifuging at 16,000 *g* for 30 min. Then, 100 μL of supernatant and 20 μL of anti-HA agarose beads were mixed with gentle rotation for 4 h at 4°C. The mixture was centrifuged at 16,000 *g* for 10 s, and the precipitate was washed 4 times with cell lysis buffer. Finally, the proteins were analyzed by Western blotting.

### Western Blotting

Cells were lysed and separated by 10% SDS-PAGE followed by transfer to PVDF membranes. The proteins of interest were probed with primary and secondary antibodies as described above. Enhanced chemiluminescence (ECL) kits were used to visualize and analyze protein bands. Films were scanned and bands were analyzed using a ChemiDoc MP Imaging System (Bio-Rad).

### Design and Synthesis of TM Peptides

The inserted peptides were confirmed to have the correct orientation because HIV TAT binds to phosphatidylinositol-(4, 5)-bisphosphate on the inner surface of the membrane (Bai et al., 2017). An HIV transactivator of transcription (HIV TAT)-linked peptide (YGRKKRRQRRR) was fused to the C-termini of the OX1R TM1 (47-67 position of amino acid), TM5 (214-235 position of amino acid) and TM7 (337-360 position of amino acid). Primary amino acid sequences of the peptides are the following: TM1, PAIYMLVFLLGTTGNGLVLWTVFYGRKKRRQRRR; TM5, VSSTTVGFVVPFTIMLTCYFFIAYGRKKRRQRRR; and TM7, LMNIFPYCTCISYVNSCLNPFLYYGRKKRRQRRR. The identity of the TM peptide sequences was confirmed by performing liquid chromatography (LC)-MS (Shimadzu2020 and Water1010). The molecular weights of TM1, 5, and 7 were 4067.95, 4209.11, and 4355.05 Da, respectively. HEK293 cells were co-transfected with OX1R-Rluc and GHSR1a-EYFP (1:3) and incubated with interference peptides corresponding to TM1 or TM5, or TM7 (4 μM) at 37°C, and BRET was detected as described above to measure the effects of interference peptides on GHSR1a/OX1R dimers.

### NFAT-RE, CRE and SRE Luciferase Reporter Assay

We detected the activity of NFAT-RE (nuclear factor of activated T-cells-response element), CRE (cAMP-response element) and SRE (serum response element) in HEK293-OX1R, HEK293-GHSR1a, and HEK293-GHSR1a/OX1R stable expression cells to study the effects of GHSR1a/OX1R heterodimers on downstream signaling. We selected three types of downstream signaling factors, specifically - NFAT-RE, CRE and SRE, which detect OX1R, GHSR1a or GHSR1a/OX1R binding to the three G protein subtypes Gαq, Gαs, and Gαi, respectively. These are useful for analyzing the effects of intracellular signal transduction pathways after GHSR1a/OX1R heterodimer formation. To perform NFAT-RE, CRE, and SRE luciferase reporter assay, the cells stably expressing GHSR1a, OX1R, or GHSR1a/OX1R were transfected with pNFAT-Luc, pCRE-Luc, or pSRE-Luc, together with pRL-Tk. The cells were starved and stimulated with orexin-A or ghrelin at 100 nM for 6 h prior to harvest at 24 h after transfection. These experiments were performed as described previously (Chen et al., 2015; Bai et al., 2017).

### Measurement of Intracellular cAMP

#### ELISA Assay for cAMP

HEK293-GHSR1a, HEK293-OX1R, and HEK293-GHSR1a/OX1R stable cell lines were cultured in 24-well cell culture plates (1–2 × 10^6^). cAMP levels were measured with a cAMP ELISA kit (Cell Biolabs, Inc., United States). The assay methods were performed as described previously (Chen et al., 2015; Liu et al., 2016).

#### BRET EPAC Biosensor for cAMP Monitoring

We also used the YFP-Epac-RLuc plasmid to measure intracellular cAMP levels (Ji et al., 2017). YFP-Epac-RLuc was transfected into HEK293-GHSR1a, HEK293-OX1R and HEK293-GHSR1a/ OX1R cells. The cells were collected and distributed in a 96-well white microplate after 24 h and cultured in HEPES-buffered phenol red-free medium for another 24 h. Cells were washed with PBS and resuspended with Dulbecco’s phosphate buffered saline (D-PBS). BRET was measured at room temperature. Cells were stimulated with agonists (ghrelin 100 nM and/or orexin-A, 100nM) for 5 min. BRET readings were collected by Tristar LB941 plate reader (Berthold technologies GmbH & Co., Germany).

### Intracellular Calcium Analysis

The stable cell lines HEK293-GHSR1a, HEK293-OX1R, and HEK293-GHSR1a/OX1R were plated at 5 × 10^4^ cells/well in 96-well microplates and cultured for 24 h. A Fluo-4 NW assay kit (Invitrogen, United States) was used as per the instructions. The solution was added to cells and incubated at 37°C for 30 min and then at 20°C for an additional 30 min (Chen et al., 2015). HEK293 cells were treated with ghrelin (100 nM) and/or orexin-A (100 nM). Fluorescence was measured with Tristar LB941 plate reader at an emission wavelength of 515 nm and excitation wavelength of 485 nm. The calcium ratio was measured by using the fluorescence value after adding the agonist taking away fluorescence value before adding agonist divided by the fluorescence value before adding the agonist.

### Gα Protein Subunit Assay

HEK-293 cells were cultured and when the cells reached ∼75% confluence, plasmids encoding EYFP (GHSR1a-EYFP, OX1R-EYFP and GHSR1a-VN173/OX1R-VC155) and Rluc (Gαs-Rluc8, Gαi2-Rluc8, and Gαq-Rluc8) fusion proteins were co-transfected. Gαs-Rluc8 or Gαq-Rluc8 or Gαi2-Rluc8 and GHSR1a-VN173 or OX1R-VC155A were also used for the negative controls. Then, cells were cultured in white 96-well cell culture plates with Poly-D-lysine-coated (fill multiwall plates with the working solution [1:19 water] and incubated for 1 h in a 37°C incubator. The solution was then removed by vacuum aspiration and allowed to surface dry. After 24 h, agonists ghrelin (100 nM) and/or orexin-A (100 nM) were added, and the cells were stimulated for 20 min. The BIFC-BRET ratio was measured in the presence of Coelenterazine h (5 μM).

### Kinetics of the GHSR1a/OX1R Heterodimer by BIFC-BRET

HEK293 cells were transfected with Rluc8-tagged (β-arrestin1-Rluc8 and β-arrestin2-Rluc8) and Venus-tagged (GHSR1a-VN173/OX1R -VC155) constructs. Two negative controls (β-arrestin1-Rluc8 or β-arrestin2-Rluc8 and GHSR1a-VN173, β-arrestin1-Rluc8 or β-arrestin2-Rluc8 and OX1R-VC155A) were also introduced. The cells were transferred to white 96-well cell (cell passage density: 5 × 10^4^/well culture plates with Poly-D-lysine-coated after 24 h. Then, the cells were cultured in HEPES-buffered phenol red-free medium. After 24 h, agonists ghrelin (100 nM) and/or orexin-A (100 nM) were added and stimulated for 20 min. The BRET ratio was measured in the presence of 5 μM Coelenterazine h. BIFC-BRET was monitored by extended BRET for 10 min to generate kinetic curves. Following the addition of orexin-A and/or ghrelin (100 nM) (Bai et al., 2017), monitoring was continued for an additional 50 min.

### Cell Proliferation Assay

Human SH-SY5Y-GHSR1a/OX1R stable cells were cultured in a 96-well plate (cell density: 4–5 × 10^4^/well). After 24 h, these cells were performed with 1, 10, and 100 nM ghrelin for another 24 h. Cell proliferation was measured using a CCK-8 (Dojindo, Japan) viability assay (Ji et al., 2017). The stable SH-SY5Y-GHSR1a, SH-SY5Y-OX1R, and SH-SY5Y-GHSR1a/OX1R cells were seeded in 96-well plates. After 24 h, all the cells were stimulated with ghrelin (100 nM) and/or orexin-A (100 nM) for 24 h. The CCK-8 assay was performed according to manufacturer’s instructions. Absorbance was measured at 450 nm after incubation. Each sample was repeated five times.

### Statistical Analysis

The data were analysed using Graph Pad Prism 6.0. All data are shown as the mean ± SEM. One-way ANOVA was used for multiple group comparisons and random single factor analysis of variance using SPSS 11.5 software. Additionally, *t*-tests were performed, and statistical significance was set at *P* ≤ 0.05. The statistical analyses were performed with SPSS (version 19.0) and the repeated measurement data were analyzed by two-way ANOVA (Repeated Measures). Statistical parameters (one-way (**Figures 5A–C**, **6B,C**, **7A,B**) or repeated measures (**Figures 6D**, **8A,D**) ANOVA) were used, and, when appropriate, with Bonferroni (comparison between all or selected groups) *post hoc* tests.

## Results

### Co-localization of GHSR1 and OX1R Was Detected Using Laser Confocal Microscopy

**FIGURE 1:**
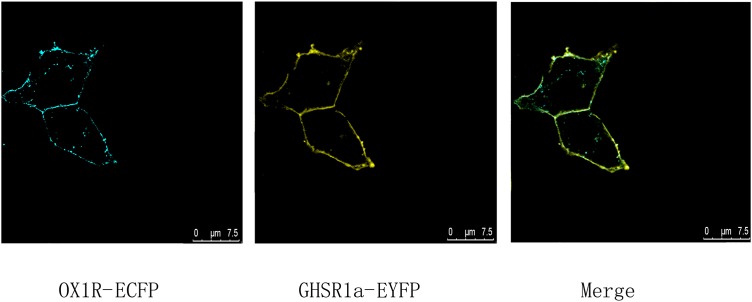
Co-expression and co-localization of GHSR1a and OX1R in HEK293 cells. Analysis of GHSR1a- and OX1R-transfected HEK293 cells using laser confocal microscopy. OX1R-ECFP (cyan) and GHSR1a-EYFP (yellow) were expressed in HEK293 cells. OX1R and GHSR1a confocal images are merged to show co-localization regions (green).

To identify co-localization of GHSR1a and OX1R in HEK293 cells, analysis of cells expressing C-terminal EYFP-tagged GHSR1a and ECFP-tagged OX1R was performed by laser confocal microscopy, the image results confirmed that GHSR1a and OX1R co-localized on membranes of HEK293 cells (**Figure 1**). The fact the cells could coexpress GHSR1a and OX1R on their membranes suggests that the two receptors might interact on the cell membranes

### BRET Detected Heterodimerization Between GHSR1a/OX1R

**FIGURE 2:**
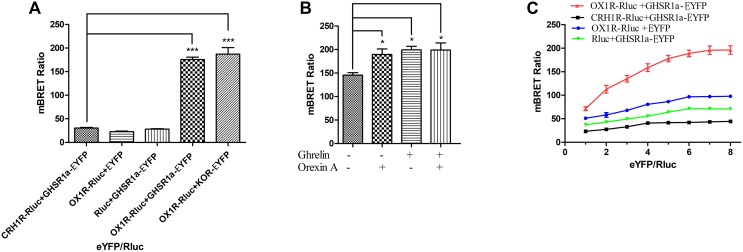
Heterodimerization of GHSR1a/OX1R examined with BRET assay in live cells. Heterodimerization of GHSR1a/OX1R was measured by BRET. HEK293 cells were transiently transfected with the donor plasmids and acceptor plasmids. Twenty-four hours after transfection, both the fluorescence and luminescence of each sample were measured prior to every experiment to confirm equal expression of Rluc while monitoring the increase in Venus expression. **(A)** BRET ratios were analyzed and are expressed as the means ± SEM (*N* = 3) of four experiments. ^∗∗∗^*p* < 0.001, OX1R-Rluc+ GHSR1a-EYFP vs. control groups (CRH1R-Rluc/GHSR1a-EYFP), as a positive control group (OX1R–Rluc/κOR-EYFP). **(B)** Effects of ghrelin and/or orexin-A on the BRET ratio. HEK293 cells were co-transfected with OX1R-Rluc and GHSR1a-EYFP plasmids (1:3). After 24 h of transfection, the Rluc substrate Coelenterazine h was added for 5 min, and the cells were treated with ghrelin (100 nM) and/or orexin-A (100 nM) or vehicle for 10 min, BRET ratios were analyzed and are expressed as the mean ± SEM of four experiments. ^∗^*p* < 0.05 compared with the control group (no treated). **(C)** BRET saturation assay. HEK293 cells were co-transfected with a constant amount of the OX1R-Rluc construct, each at 0.15 μg/well, and increasing amounts of the EYFP construct (0.15–1.2 μg/well). Calculated BRET ratios were plotted relative to total fluorescence/luminescence ratios, and the data were analyzed by non-linear regression curve fitting (one site–specific binding) using GraphPad Prism software. BRET ratios were analyzed and are expressed as means ± SEM (*N* = 3) of four experiments.

To determine whether GHSR1a/OX1R can form a heterodimer, a BRET test based on luminescence and fluorescence detection was performed. In this experiment, the energy donor (Rluc fusion protein) and energy receptor (EYFP fusion protein) transfection ratio was 1:3. As shown in **Figure 2A**, the mBRET ratio of OX1R-Rluc to GHSR1a-EYFP (190 ± 0.010) was lower than that of the positive control group OX1R–Rlu/κOR-EYFP (Chen et al., 2015) (mBRET ratio 195 ± 0.030), but there was not a significant difference between the two, and the mBRET ratio was higher than that of the negative control group (CRH1R-Rluc/GHSR1a-EYFP) (Navarro et al., 2015) (mBRET ratio: 37.5 ± 0.010), with a significant difference between the two. Cells expressing GHSR1a-EYFP with Rluc or OX1R-Rluc with EYFP were also used as a negative control. The results showed that BRET could occur between the GHSR1a/OX1R in cells. These results suggest that heterodimerization of GHSR1a/OX1R may not require ligand binding.

BRET was also used to determine whether ligands of OX1R/GHSR1a induce heterodimerization. As shown in **Figure 2B**, the OX1R/GHSR1a dimer is a constitutive dimer, but ghrelin or orexin-A enhanced the interaction between OX1R and GHSR1a such that their addition caused the formation of many additional dimers.

To investigate the heterodimerization of GHSR1a/OX1R, BRET saturation assay was used to detect their interactions in HEK293 cells. Living Cells were co-transfected with increasing amounts of acceptor (GHSR1a-EYFP or EYFP) and constant amount of the donor (OX1R-Rluc, CRH1R-Rluc or Rluc). As shown in **Figure 2C**, the BRET ratio between GHSR1a-EYFP and OX1R-Rluc increased with the increasing amounts of acceptor expression of GHSR1a-EYFP, until a plateau was reached (**Figure 2C**). However, in the negative control group, CRH1R-Rluc and GHSR1a-EYFP, increasing expression of GHSR1a-EYFP resulted in a low ratio. This suggests a non-specific linear relationship between them. This is consistent with a previous report that BRET signals are low when OX1R-Rluc and GHSR1a-EYFP are co-transfected. These results indicate that OX1R-Rluc and GHSR1a-eGFP can form heterodimers (Navarro et al., 2015; Bai et al., 2017).

### FRET Assay

**FIGURE 3:**
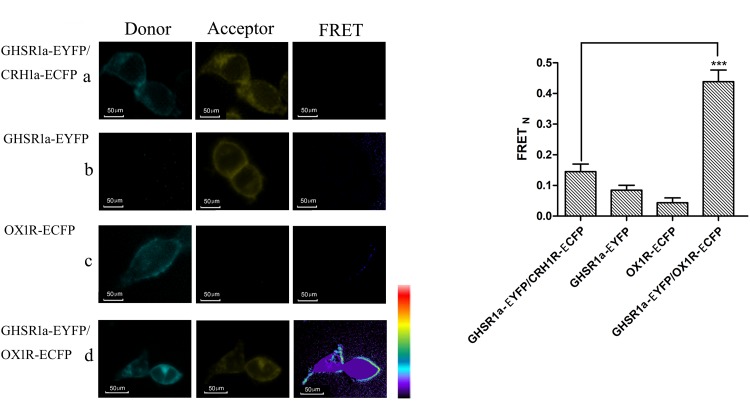
Förster resonance energy transfer assay to detect the dimerization of GHSR1a/OX1R. **(A)** FRET imaging of constitutive GHSR1a/OX1R heteromeric interactions in living cells. HEK293 cells were transiently transfected with plasmids encoding **(a)** as a negative control group, CRH1R-ECFP and GHSR1a-EYFP. **(b)** GHSR1a-EYFP (acceptor), **(c)** OX1R-ECFP (donor), **(d)** OX1R-ECFP and GHSR1a-EYFP. Left panels, ECFP; center panels, EYFP; right panels, corrected FRET. **(B)** Normalized FRET values, calculated as described in Experimental Procedures. The data represent mean ± SEM (*N* = 3) of four independent experiments. Statistical analysis was performed using one-way ANOVA followed by Tukey’s multiple comparison *post hoc* test. ^∗∗∗^*p* < 0.001 vs. the negative groups CRH1R-ECFP and GHSR1a-EYFP.

Fluorescence resonance energy transfer was used to analyze the interaction between GHSR1a/OX1R. In these experiments, HEK293 cells were transfected with (a) CRH1R-ECFP and GHSR1a-EYFP, (b) GHSR1a-EYFP, (c) OX1R-ECFP, or (d) OX1R-ECFP and GHSR1a-EYFP. In these images, interaction sites are marked yellow (**Figure 3A**). In individually transfected cells and control cells containing GHSR1a-EYFP and CRH1R-ECFP, lower FRET signals were observed. However, a FRET signal was detected doubly co-transfected with GHSR1a-EYFP and OX1R-ECFP, further indicating that GHSR1a/OX1R may form dimers (**Figure 3B**).

### Heterodimerization of GHSR1a/OX1R Determined by Co-immunoprecipitation (Co-IP)

**FIGURE 4:**
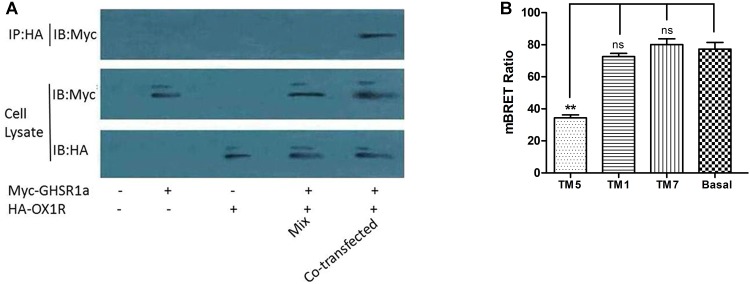
The dimerization of GHSR1a/OX1R and effects of interfering peptides on GHSR1a/OX1R heterodimers. **(A)** HEK293 cells were either not transfected (mock) or transfected with HA–OX1R, Myc–GHSR1a or both (co-transfection). As negative control, samples containing either HEK293 HA–OX1R cells or HEK293 Myc–GHSR1a cells were mixed (Mix). Cell lysates were immunoprecipitated with anti-HA agarose beads and immunoblotted with anti-Myc antibody (upper panel). Cell lysates were examined by immunoblotting with either an anti-Myc or anti-HA antibody (lower panels). The data represent means ± SEM (*N* = 3) of four independent experiments. **(B)** HEK293 cells were co-transfected with OX1R-Rluc and GHSR1a-EYFP (1:3) and incubated at 37°C for 2 h with HIV TAT–fused TM peptides (10 μM) corresponding to TM1, TM5, or TM7 of OX1R. BRET ratios were analyzed and are expressed as the means ± SEM (*N* = 3) of four experiments (one-way analysis of variance; ns, not significant, *p* > 0.05; ^∗∗^*p* < 0.01 vs. control group).

Co-immunoprecipitation analysis was used to identify the interaction between OX1R and GHSR1a in the formation of a heterodimer. After co-expression in HEK293 cells of HA-OX1R/Myc-GHSR1a, using anti-Myc antibodies to do immunoprecipitation resulted in the presence of anti-HA immunoreactivity. SDS-PAGE demonstrated the presence of bands identified by the Myc antibody (**Figure 4A**), consistent with the monomeric form of HA-OX1R. This result showed that GHSR1a/OX1R could form heterodimers, when expressed simultaneously. When Myc-GHSR1a or HA-OX1R were singly overexpressed or placed in mixed samples, the Co-IP results were similar to those for the negative control.

### TM1, 5, and 7 Provide the GHSR1a/OX1R Dimer Interface

Transmembrane domains (TMs) are important for the formation of head-to-head interfaces in class A GPCR dimers (Johnston et al., 2012; Meng et al., 2014; Cordomi et al., 2015; Xue et al., 2015). To identify dimerization interfaces among the seven TM domains of GHSR1a/OX1R, the effects of cell-penetrating interference peptides containing the sequence of the hydrophobic transmembrane helices on GHSR1a/OX1R heterodimerization were examined by BRET.

As shown in **Figure 4B**, TM 5 significantly reduced GHSR1a/OX1R dimer BRET signals by 50–60%. Thus, peptides corresponding to TM 5 can disrupt the formation of GHSR1a/OX1R heterodimers, further suggesting the involvement of TM 5 at the GHSR1a/OX1R heterodimer interface. Notably, TM1 and TM7 had little effect on BRET signals, indicating that these peptides do not induce significant conformational changes or are less important for GHSR1a/OX1R dimerization *in vitro*.

### Ghrelin Increases CRE-luc, NFAT-luc and SRE-luc Activity in HEK293-GHSR1a/OX1R Cells

**FIGURE 5:**
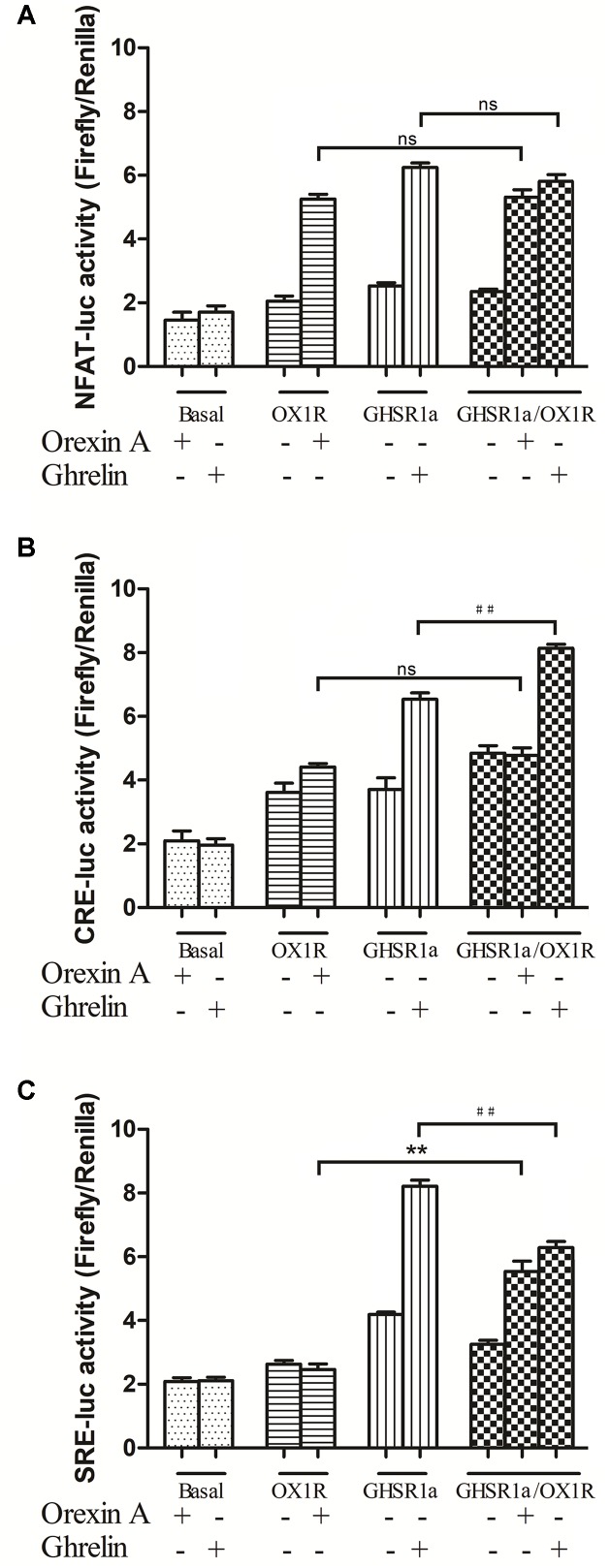
Effects of GHSR1a/OX1R heterodimerization on NFAT, CRE and SRE activities. Twenty-four hours after transfection with pNFAT-luc **(A)**, pCRE-luc **(B)**, and pSRE-luc **(C)** together with pRL-TK, HEK293-GHSR1a, HEK293-OX1R, and HEK293-GHSR1a/OX1R cells were stimulated with ghrelin (100 nM) and/or orexin-A (100 nM) for 6 h prior to harvest. Firefly and Rluc activities were assay using a Dual-Luciferase Reporter Assay System, and the ratios of firefly to Rluc luminescence were calculated. The data represent the means ± SEM (*N* = 3) of at least four independent experiments. Statistical analysis was performed using one-way ANOVA followed by Tukey’s multiple comparison *post hoc* test. ^∗∗^*p* < 0.01, cells co-expressing both GHSR1a and OX1R treated with orexin-A vs. cells expressing OX1R alone; ^##^*p* < 0.01, HEK293 cells co-expressing GHSR1a and OX1R treated with ghrelin vs. cells expressing GHSR1a alone. ns, not significant, *p* > 0.05.

To analysis the effect of GHSR1a/OX1R dimers on downstream signaling, the activity of NFAT-RE, SRE, and CRE in HEK293-OX1R, HEK293-GHSR1a and HEK293-GHSR1a/OX1R cells were assessed respectively, as shown in **Figure 5**.

In HEK293-GHSR1a cells, the activity of NFAT-RE and CRE increased significantly with ghrelin exposure (*p* < 0.01). The results suggested that GHSR1a coupled to the Gαq and Gαs subtypes. Interestingly, Gαi-coupled receptors inhibited adenylyl cyclase (AC), and through the βγ subunits, activated the ERK pathway via SRE-luc in the HEK293 GHSR1a cells with ghrelin, the results showed that GHSR1a coupled to the Gαi and the βγ subunits. The SRE-luc activity of GHSR1a/OX1R cells treated with orexin A was increased (*p* < 0.01), compared with HEK293 OX1R cells. The ghrelin treatment of HEK293 cells co-expressing both receptors further decreased SRE-luc activity, compared with HEK293 GHSR1a cells. The intracellular CRE-luc activity of GHSR1a/OX1R cells treated with ghrelin was increased (*p* < 0.01), and treatment of HEK293 GHSR1a/OX1R cells with orexin-A had no effect on CRE–luc activity. The results showed that heterodimerization of GHSR1a/OX1R increased CRE activity when ghrelin was added to HEK293-GHSR1a and OX1R cells. This which further confirms that the GHSR1a/OX1R heterodimer promotes the Gαs signaling pathway.

### Detection of Intracellular cAMP

**FIGURE 6:**
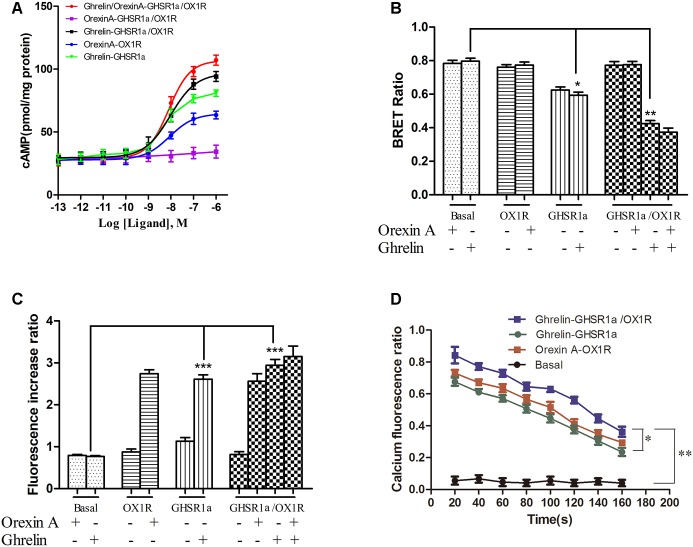
Effect of GHSR1a/OX1R heterodimerization on intracellular second messengers. **(A)** ELISA assay for cAMP. HEK293-GHSR1a, HEK293-OX1R, and HEK293-GHSR1a/OX1R cells were stimulated with forskolin (10 μM) in the absence or presence of various concentrations of orexin-A (0.001–1000 nM) and/or ghrelin (0.001–1000 nM). Intracellular cAMP concentrations were measured using an ELISA assay. Values represent the mean ± SEM (*N* = 3) of three measurements from three independent experiments. The curve was fitted using nonlinear regression [log (agonist) vs. response – variable slope] in Prism 5.0. **(B)** BRET EPAC biosensor for cAMP monitoring. Measurement of BRET signals in HEK293 cells co-expressing the cAMP biosensor and GHSR1a, OX1R, or GHSR1a/OX1R and stimulated with ghrelin and/or orexin-A. ^∗^*p* < 0.05; ^∗∗^*p* < 0.01. The data are expressed as the mean ± SEM (*N* = 3) of four independent experiments. **(C)** Intracellular calcium assay. HEK293 cells stably expressing GHSR1a, OX1R, or GHSR1a/OX1R were treated with ghrelin (100 nM) or orexin-A (100 nM). The fluorescence intensity of intracellular calcium was recorded 20 s after stimulation using a Fluo-4 NW Calcium Assay Kit. Untransfected cells were used as a control. The results are the mean ± SEM (*N* = 3) of at least four independent experiments. Statistical analysis was performed using one-way ANOVA followed by Tukey’s multiple comparison *post hoc* test. ^∗∗∗^*p* < 0.001 vs. basal groups. **(D)** Time-dependent activation of intracellular calcium by ghrelin or orexin-A in HEK293-GHSR1a, OX1R, or GHSR1a/OX1R cells. The intracellular calcium concentration was detected as described above. The statistical analyses were performed with SPSS (version 19.0). It was found that there are differences in different time results, and there are differences among different groups at different times (20, 80, and 140 s). The results of the comparison between the two groups showed that there was a statistical difference between the basal group and the other three groups (*P* < 0.01), and there was a statistical difference between double-transfected cells group and single-transfected cells group (*P* = 0.042, *P* = 0.017).

To detect whether GHSR1a/OX1R dimerization leads to change in signaling pathways, intracellular cAMP concentrations were measured using an ELISA assay kit. First, we compared cAMP accumulation in HEK-GHSR1a, HEK293-OX1R and HEK293-GHSR1a/OX1R cells. In the HEK-293 GHSR1a/OX1R cells with ghrelin stimulation, cAMP levels were higher than in HEK293 GHSR1a or HEK293-OX1R cells significantly (**Figure 6A**). However, when treated with orexin-A, BRET signals in HEK293-GHSR1a/OX1R cells did not differ from those in HEK293-OX1R cells (**Figure 6B**), suggesting that orexin-A cannot enhance the cAMP level through the GHSR1a/OX1R heterodimer. The results suggest that GHSR1a/OX1R may strengthen Gαs coupling, when GHSR1a /OX1R are expressed with ghrelin stimulation.

To confirm this finding, we also used the cAMP BRET biosensor method to determine whether the heterodimer affects cAMP accumulation. The sensor consists of an N-terminal-truncated variant of EPAC tagged with Rluc and YFP at the N and C termini, respectively (Barak et al., 2008; Ji et al., 2017). After binding of the cAMP, this change in conformation leads to a decrease in BRET that presumably increases the distance between the donor and acceptor. The reduction in the BRET signal with FSK is consistent with its role as a stimulator that promotes cAMP production (Barak et al., 2008). After treatment with ghrelin, BRET signals were lower in HEK293-GHSR1a/OX1R cells than in HEK293- GHSR1a and HEK293-OX1R cells significantly (**Figure 6B**). However, after treatment with orexin-A, BRET signals in HEK293-GHSR1a/OX1R cells did not differ from those in HEK293-OX1R cells (**Figure 6B**), suggesting that through GHSR1a/OX1R heterodimers ghrelin induces a cAMP-response element signaling, but orexin-A cannot enhance the cAMP level through the GHSR1a/OX1R heterodimer.

### Detection of Intracellular Calcium Concentration in HEK293-GHSR1a/OX1R Cells

Calcium is the downstream second messenger of the Gαq subtype of the G protein signaling pathway, and the detection of intracellular calcium facilitates elucidation of the effects of the GHSR1a/OX1R dimer on the intracellular signal transduction pathway. The results are shown in **Figures 6C,D**. The calcium content in HEK293-OX1R cells (2.8 ± 0.2) was significantly increased compared with that in the basal group (0.58 ± 0.08) under the action of orexin-A. The concentration of calcium in HEK293-GHSR1a cells (2.8 ± 0.05) was significantly different from that in the empty vector group (0.54 ± 0.05). In addition, the statistical analyses (**Figure 6D**) were performed with SPSS (version 19.0). It was found that there are differences in different time results, and there are differences among different groups at different times (20, 80, and 140 s). The results of the comparison between the two groups showed that there was a statistical difference between the basal group and the other three groups (*P* < 0.01), and there was a statistical difference between double-transfected cells group and single-transfected cells group (*P* = 0.042, *P* = 0.017).

However, the calcium levels in HEK293-GHSR1a/OX1R cells did not differ from those in HEK293-GHSR1a or HEK293-OX1R cells. Based on these results, the GHSR1a/OX1R dimer is not associated with the G protein binding of the Gαq subtype, and the GHSR1a/OX1R dimer alters the signal transduction pathway of the original monomer.

### Identification of a G-protein Coupling to GHSR1a/OX1R Heterodimers Using BiFC-BRET

GHSR1a/OX1R heterodimers can exhibit a signaling that is different from that of their monomers. To survey interactions between GHSR1a/OX1R heterodimers and Gα, BIFC-BRET assay were performed (Cai et al., 2017).

**FIGURE 7:**
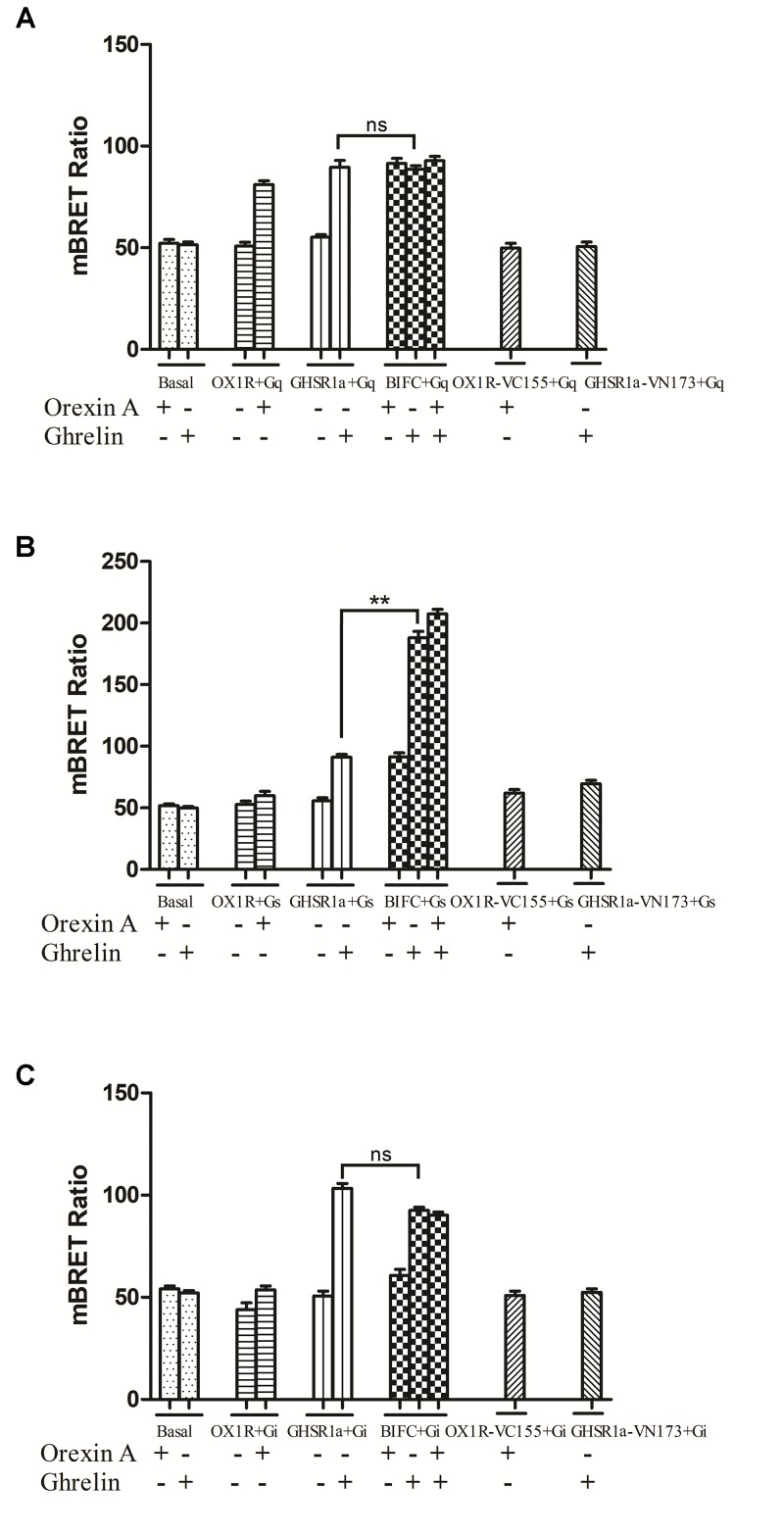
Bioluminescence resonance energy transfer analysis of the effects of GHSR1a/OX1R heterodimerization on Gα protein subunit activation. HEK293 cells expressing GHSR1a, OX1R or BIFC (GHSR1a/OX1R) were stimulated with 100 nM ghrelin and/or 100 nM orexin-A for 20 min, and analysis of the interaction between GHSR1a/OX1R heterodimers and **(A)** Gαq-Rluc8, **(B)** Gαs-Rluc8, and **(C)** Gαi2-Rluc8 was performed using BRET. ^∗∗^*p* < 0.01, HEK293 cells co-expressing GHSR1a/OX1R stimulated with ghrelin (100 nM) vs. HEK293 cells expressing GHSR1a only;. The data points represent the mean ± SEM (*N* = 3) of four independent experiments. ns, not significant, as determined by two independent samples *t*-test.

As shown in **Figure 7**, when GHSR1a and OX1R were co-expressed, changes in the BRET ratio between BiFC (GHSR1a-VN173 and OX1R-VC155) and Rluc8-tagged Gαq (**Figure 7A**), Gαs (**Figure 7B**), or Gαi2 (**Figure 7C**) were observed. In response to corresponding ligands, the expression of Rluc8-labeled Gαi2 (Rluc8-Gαi2) and BiFC (GHSR1a/OX1R) in HEK293 cells did not undergo significant changes in terms of the BRET ratio. In the HEK293-GHSR1a/OX1R cells after agonist stimulation, the BRET ratio between BiFC (GHSR1a-VN173 and OX1R-VC155) and Rluc8-tagged Gαq was smaller than that expressing GHSR1a or OX1R alone in cells.

More interestingly, the BRET ratio between BiFC (GHSR1a-VN173 and OX1R-VC155) and Rluc8-tagged Gαs showed a significant increase after ghrelin stimulation compared with that of cells expressing GHSR1a alone. The results indicate that GHSR1a/OX1R heterodimers activate Gαs but not Gαi2 or Gαq and that ghrelin induces a Gαs signaling via GHSR1a/OX1R heterodimers (**Figure 7B**).

### β-arrestin Assay of GHSR1a/OX1R Heterodimerization

**FIGURE 8:**
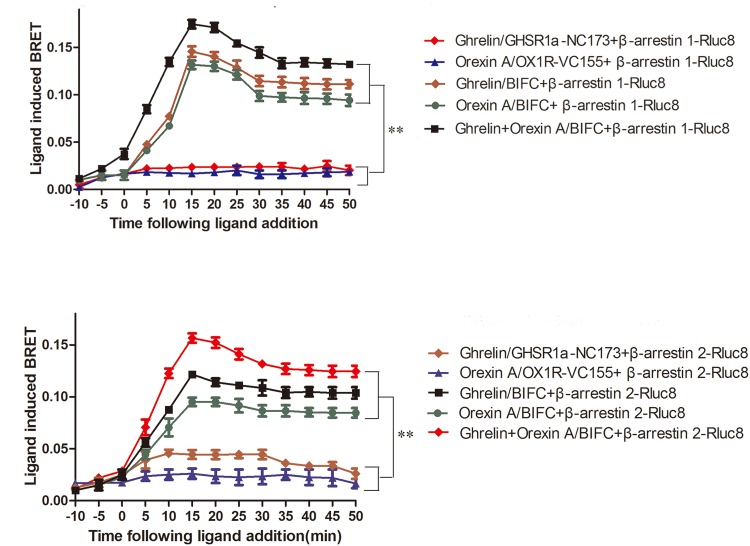
Characterization of β-arrestin recruitment to GHSR1a/OX1R heterodimers. HEK293 cells transiently co-expressing BIFC (GHSR1a-VN173/OX1R-VC155) and **(A)** β-arrestin1-Rluc8 or **(B)** β-arrestin2-Rluc8 were assessed using BIFC-BRET to generate kinetic curves. Following the addition of 100 nM ghrelin and/or 100 nM orexin-A, the test continued for 50 min. The data points represent the mean ± SEM (*N* = 3) of four independent experiments. The statistical analyses were performed with SPSS (version 19.0). The results of taking time 15, 25, and 35 min to analyze the results of four measurements show that there are statistical differences in different times. There was no significant difference between the control groups (two groups) and the experimental groups (three groups). The difference between the control groups and the experimental groups was statistically significant (*P* < 0.01).

When HEK293 cells expressing β-arrestin1-Rluc8 and BIFC (GHSR1a-VN173 and OX1R-VC155) were treated with ghrelin (100 nM) and/or orexin-A (100 nM), a strong and increasing ligand induced BRET signal was noted, indicating that β-arrestin1 is raised as a result of activated GHSR1a/OX1R heterodimers (**Figure 8A**). Ghrelin or orexin-A treatment of cells transfected with BIFC and β-arrestin1-Rluc8 increased BRET value with a reduced magnitude but a similar kinetic profile. No changes in BRET were observed with the negative controls (β-arrestin 1-Rluc8 and GHSR1a-VN173, or β-arrestin 1-Rluc8 and OX1R-VC155). However, incubation of HEK293 cells co-expressing GHSR1a/OX1R with ghrelin or and orexin-A increased BRET value, indicating the proximity of these heteromeric complexes that recruit β-arrestin1 (**Figure 8A**). Similarly, ligand-dependent recruitment of β-arrestin2 was seen in these heterodimer complexes (**Figure 8B**). The statistical analyses (**Figures 8A,B**) were performed with SPSS (version 19.0). The results of taking time 15, 25, and 35 min to analyze the results of four measurements show that there are statistical differences in different times. There was no significant difference between the control groups (two groups) and the experimental groups (three groups). The difference between the control groups and the experimental groups was statistically significant (*P* < 0.01). The results indicated that GHSR1a/OX1R heterodimer does not change recruitment of β-arrestins.

### Ghrelin Enhances SH-SY5Y Cell Proliferation

**FIGURE 9:**
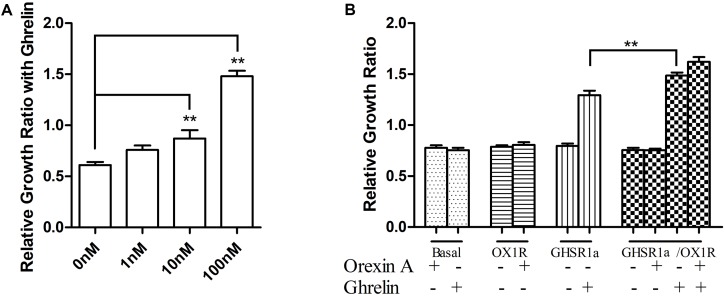
Proliferation of stable cell groups in response to ghrelin. **(A)** Dose-dependent effects of ghrelin on stable SH-SY5Y-GHSR1a/OX1R cell proliferation. Cell proliferation was measured using a CCK-8 proliferation/viability assay. Stable cells that were not stimulated with ghrelin were used as the control. ^∗∗^*p* < 0.01, cells co-expressing both GHSR1a/OX1R and treated ghrelin vs. control. **(B)** The stable SH-SY5Y-GHSR1a, SH-SY5Y-OX1R, and SH-SY5Y-GHSR1a/OX1R cells or SH-SY5Y cells were cultured in a 96-well plate and starved overnight in serum-free medium. Then, cells were stimulated with 100 nM ghrelin or 100 nM orexin-A or 100 nM ghrelin/orexin-A. Cell proliferation was measured as above. SH-SY5Y cells (Untransfected cells) were used as the control. The data points represent the mean ± SEM (*N* = 3) of four independent experiments. ^∗∗^*p* < 0.01, cells co-expressing both GHSR1a/OX1R treated with 100 nM ghrelin vs. control.

In the above experiments, we observed that GHSR1a/OX1R may heterodimerize. To discuss the GHSR1a/OX1R dimerization effect on vitality of cells, we examined the GHSR1a/OX1R heterodimerization effect on cell proliferation. As shown in **Figure 9A**, ghrelin had a significant effect on the proliferation of SH-SY5Y-GHSR1a/OX1R cells, and this functional outcome of GHSR1a/OX1R co-expression followed a dose-dependent pattern, reaching significance (*p* < 0.01) at 100 nM (**Figure 9A**). Interestingly, the cell viability of the SH-SY5Y-GHSR1a/OX1R group was also higher than the SH-SY5Y-GHSR1a group treated with ghrelin (**Figure 9B**). These data indicate that activation of GHSR1a/OX1R heterodimers could increase proliferation of the SH-SY5Y neuroblastoma cell.

## Discussion

GPCRs are well known to exist as monomeric entities that can form dimers and/or oligomers when expressed in a heterologous cell system, as well as in native tissues. For example, human muscarinic M1 receptor is present on the basolateral surface of cells as a 75:25 mixture of receptor monomers and dimers/oligomers (Pediani et al., 2016; Bai et al., 2017). The proportion of APJ monomers, dimers, and oligomers is ∼40%, ∼36%, and ∼24%, respectively (Cai et al., 2017). However, the proportion of these forms can be regulated by interactions with ligands, and these effects have major implications for receptor function and behavior.

GPCR dimerization *in vivo* and *in vitro* has been demonstrated using a range of techniques, including Co-IP, resonance energy transfer and protein fragment complementation assay (Borroto-Escuela et al., 2013; Gomes et al., 2016). GHSR1a and OX1R are found co-expressed in many brain regions such as the cortex, hypothalamus, pituitary, hippocampus, amygdala, ventral tegmentum and nucleus raphe. We speculated that receptor coexpression in the same neurons can lead to interactions between GHSR1a and OX1R by heterodimers.

Here, BRET, FRET, and BiFC analysis and traditional Co-IP data provide solid evidence for the formation of functional GHSR1a/OX1R heterodimers. Additionally, we examined the interacting interfaces of GHSR1a/OX1R dimers using interference peptides and BRET. Through the study of interfering peptides’ effects on interactions between GHSR1a/OX1R, we discovered that TM5, but not TM1 or TM7, could bind to OX1R, showing that TM5 is involved in the heterodimer interface formation. Most likely, two of these form the dimer in the inactive state. Furthermore, the main dimer interface in the active state requires further investigation. These findings suggest that these interfering peptides have therapeutic potential, as they can disrupt dimerization and influence receptor function. Indeed, a peptide derived from TM6 of β2-adrenergic receptor (β2AR) disrupts dimer formation and decreases receptor function (Chen et al., 2015; Koroglu and Akten, 2015). Thus, identification of a peptide that interferes with the D1R-D2R interaction and has antidepressant activity may provide a new therapeutic strategy for the treatment of major depression (Pei et al., 2010).

Heteromerization of GPCRs is an important mechanism that can regulate receptor function. Receptor–receptor interactions potentially stabilize specific conformations and lead to coupling with discrete effectors, resulting in heterodimer-specific signal transduction (Kern et al., 2012; Ferré et al., 2014; Franco et al., 2016). For example, Chen et al. have showed that heterodimerization of human OX1R and κOR promotes protein kinase A and cAMP-response element binding protein signaling via a Gαs-mediated mechanism (Chen et al., 2015). Jiang et al. (2006) have shown a molecular mechanism for the synergistic effect of dopamine receptor D1-GHSR1a dimerization on cAMP accumulation (Jiang et al., 2006). In the ventral tegmental area, OX1R and corticotropin-releasing factor (CRF) receptor heterodimers serve as targets for cocaine and promote long-term disruption of negative crosstalk between orexin-A and CRF (Navarro et al., 2015; Bai et al., 2017). Heteromultimerization of cannabinoid (CB) 1 receptor and OX1R generates a unique complex, in which both protomers are regulated by orexin-A (Ward et al., 2011; Bai et al., 2017). Rediger et al. (2009) have demonstrated GHSR1a-melanocortin-3 receptor (MC3R) dimers using FRET. Typical signaling through MC3R involves a Gαs pathway leading to cAMP accumulation (Rediger et al., 2009).

In the present study, using both BRET and FRET technology, we confirmed that GHSR1a/OX1R form heterodimers. BRET analysis also indicated that ghrelin or orexin-A treatment had little effect on the interaction between GHSR1a/OX1R. To further study whether heterodimerization leads to signaling alterations and allosteric signaling, we researched the effects of GHSR1a/OX1R heterodimerization on G-protein subunit coupling. Surprisingly, although it has been previously reported that GHSR1a/OX1R mainly couple with the major Gαq receptors, we found that the BRET ratio between BiFC (GHSR1a-VN173 and OX1R-VC155) and Rluc8-tagged Gαs was significantly increased after ghrelin or ghrelin/orexin-A stimulation compared with that of cells expressing GHSR1a or OX1R alone, suggesting that Gαs could be activated by the dimer. By contrast, activation of Gαq and Gαi2 was not found in the cells expressing GHSR1a/OX1R, which suggests that GHSR1a/OX1R heterodimerization upregulated Gαs-protein coupling and leads to a Gαs signaling and asymmetric allosteric signaling.

In recent years, there has been controversy over whether GPCRs present during physiological function as monomers (Whorton et al., 2008), dimers (Fung et al., 2009; Han et al., 2009; Franco et al., 2016), or even larger oligomers (Gurevich and Gurevich, 2008; Bouvier and Hebert, 2014). An obvious functional advantage of dimers, which are easier to understand in heterologous contexts, is that they can interact through allosteric interactions that may or may not be dependent on ligand occupancy. In this context, GPCR oligomers can function as allosteric machines (Kenakin, 2012; Ferré et al., 2014; Moreno et al., 2016). For example, one ligand-occupied protomer in a heterodimer can act as an allosteric modulator for the other protomer, affecting ligand binding and/or signaling conduction (Muller et al., 2012). The findings of our study are consistent with this notion. GHSR1a/OX1R heterodimers can induce significant changes in downstream signaling with ghrelin stimulation, indicating that heterodimerization of GHSR1a/OX1R is a complex mechanism regulating receptor function, especially in asymmetric allosteric and two ligand regulation. The study results showed that allosteric communication between the dimer could select the downstream signal pathway.

Furthermore, second messenger concentrations have been measured. The results show that cAMP accumulation in double-transfected cells treated with ghrelin or ghrelin/orexin-A was significantly greater than in single-transfected cells. The cAMP levels increased in a dose-dependent manner. The results indicate that GHSR1a/OX1R may strengthen Gαs coupling, when GHSR1a/OX1R are expressed together. The Ca^2+^ levels were also measured after stimulation with ghrelin or orexin-A observed no significant difference in any of the three cell types. This suggests that the distinct conformations are mainly caused by receptor heterodimerization, which increases the activation of Gαs (Chen et al., 2015).

The effects on the Gαs/Gαq/Gαi signaling pathways of GHSR1a/OX1R heterodimers were confirmed by detection of signals downstream from CRE, NFAT-RE and SRE. The results showed that NFAT-RE and SRE activity in single-transfected cells was higher than in double-transfected cells. In double-transfected cells with ghrelin stimulation, CRE activity was significantly higher than in ghrelin-treated single-transfected cells, suggesting that CRE activity is induced via the Gαs/cAMP/cAMP-response element signaling pathway upon co-expression of the two receptors. The increasing activity of CRE provide further evidence that dimerization of GHSR1a/OX1R, when co-expressed, leads to an increase in Gαs coupling. Taken together, we suggest that allosteric signaling through GHSR1a/OX1R heterodimers represents a Gαs/cAMP /cAMP-response element signaling pathway. In this way, rearrangements of structure can elicit a change in signaling cascades within GPCR dimers and demonstrate an important role in structural rearrangements that confirm downstream signaling is mediated by interaction between GHSR1a and OX1R. Although it is difficult to transfer the mechanism of overexpression research in the HEK 293 cells to the endogenous expression receptor in the cell system and *in vivo*, the presence and functional relevance of GPCR dimers or higher oligomers is usually accepted. GPCRs form dimers in heterologous cells, resulting in an apparent plethora of functional consequences. Further research would be to investigate to the physiological functions for GPCR Dimers in native tissue.

To evaluate whether dimerization of GHSR1a/OX1R affects cellular functions (for example, cell viability), and to investigate whether the neuronal cells that coexpress GHSR1a and OX1R are characterized by cellular functions, we selected the SH-SY5Y neuroblastoma cell line that generated SH-SY5Y cells that stably express GHSR1a and OX1R. Cell proliferation was investigated. After treatment with ghrelin, the proliferation of SH-SY5Y GHSR1a and OX1R cells was significantly higher than that of SH-SY5Y GHSR1a or SH-SY5Y OX1R cells alone. Furthermore, ghrelin induced the proliferation of human SH-SY5Y GHSR1a and OX1R cells in a dose-dependent manner. These results indicate that ghrelin plays a role by increasing the proliferation of SH-SY5Y GHSR1a and OX1R cells.

The results of the present study are consistent with those of a series of previous experiments indicating that structural rearrangements of GPCRs can influence cell signaling, in that ghrelin stimulation of GHSR1a/OX1R heterodimers induced great changes in downstream signaling, indicating that herterodimerization of GHSR1a/OX1R is an underlying mechanism by which receptor function is complex, especially upon asymmetric allosteric regulation.

## Conclusion

This study reports that GHSR1a forms heterodimers with OX1R. We showed that ghrelin stimulation of cells expressing GHSR1a/OX1R heterodimers led to activation of Gαs proteins. Stimulation of GHSR1a/OX1R heterodimers with orexin-A did not alter GPCR interactions with Gα protein subunits. GHSR1a/OX1R heterodimers induced signaling pathway activity of Gαs/protein kinase A pathway, including increasing cAMP-response element luciferase reporter activity and cAMP levels. In addition, ghrelin induced a higher proliferation rate in SH-SY5Y cells than control. This suggest that ghrelin through GHSR1a/OX1R heterodimers reveals a Gαs–cAMP-cAMP response element binding protein signaling *in vitro*. However, translation of *in vitro* observations for GHSR1a/OX1R heterodimer to native tissues is required in the future to confirm that heterodimers exist *in vivo*. The roles of GHSR1a/OX1R heterodimers are needed to further investigate the physiological functions *in vivo* experiments, such as using *in situ* proximity ligation assay, and time-resolved FRET method.

## Author Contributions

BB and JC designed the study and revised the manuscript. QX, CY, BJ, YP, RZ, and YJ conducted most of the experiments. QX, XC, and JC wrote the paper. PW, XC, CW, and BC analyzed and interpreted the data. All authors reviewed and approved the manuscript.

## Conflict of Interest Statement

The authors declare that the research was conducted in the absence of any commercial or financial relationships that could be construed as a potential conflict of interest.

## Abbreviations

BRET: bioluminescence resonance energy transfer
EYFP: enhanced yellow fluorescent protein
FRET: Förster resonance energy transfer
GHSR1a: growth hormone secretagogue receptor 1α
GPCRs: G-protein-coupled receptors
HEK: human embryonic kidney
OX1R: orexin type 1 receptor
Rluc: Renilla luciferase
